# Editorial: Protecting the code: DNA double-strand break repair pathway choice

**DOI:** 10.3389/fgene.2022.993889

**Published:** 2022-08-12

**Authors:** Jenny Kaur Singh, Sylvie M. Noordermeer, Judit Jimenez-Sainz, David G. Maranon, Matthias Altmeyer

**Affiliations:** ^1^ Department of Human Genetics, Leiden University Medical Center, Leiden, Netherlands; ^2^ Institut Curie, PSL University, CNRS, Orsay, France; ^3^ Oncode Institute, Utrecht, Netherlands; ^4^ Department of Therapeutic Radiology, Yale University School of Medicine, New Haven, CT, United States; ^5^ Department of Environmental and Radiological Health Sciences, Colorado State University, Fort Collins, CO, United States; ^6^ Department of Molecular Mechanisms of Disease, University of Zurich, Zurich, Switzerland

**Keywords:** genome stability, chromatin, DNA repair, DNA replication, genome editing, cancer

The genetic information in our cells is constantly challenged by several sources that can cause DNA damage. Amongst the various lesions that can occur in the genome, DNA double-strand breaks (DSBs) are considered the most dangerous (Scully et al., 2019). Cells combat DSBs by activation of the DNA damage response (DDR), a complex evolutionary conserved cellular network that senses, signals and repairs DNA breaks, while coordinating DNA repair with chromatin regulation, gene expression, and cell cycle progression (Ciccia and Elledge, 2010). Defects in the DDR can lead to devastating diseases such as immune disorders and cancer (Jackson and Bartek, 2009). In addition, the targeted generation of DSBs can be exploited for CRISPR-mediated genome editing and gene therapy (Yeh et al., 2019; Nambiar et al., 2022). DSB repair is, therefore, one of the most critical tasks a cell must pursue to maintain genome integrity, and malfunction of this process has important clinical implications.

This Research Topic is focused on *Protecting the code: DNA double-strand break repair pathway choice* and features 18 articles that reflect the complexity of cellular processes that determine DNA repair pathway choice. It consists of topical reviews as well as original research and methods articles focusing on key DNA repair mechanisms, including the main DSB repair pathways non-homologous end-joining (NHEJ) and homologous recombination (HR), and on techniques to study these pathways and elucidate their relevance for human health and disease.

DSB repair pathway choice relies on multiple regulatory layers that can respond to environmental and cell-intrinsic cues (Chapman et al., 2012; Krenning et al., 2019). Amongst the latter are non-B DNA structures, which are formed at particular sequences (e.g., at repetitive regions or at common fragile sites) and can exist in the form of G-quadruplexes (G4) and RNA-DNA hybrids (R-loops). In the article by Camarillo et al. an update and perspective is provided on the tight interconnection between G-quadruplexes and R-loops and their emerging role as roadblocks for DNA end-resection during DSB repair by HR. Regarding the temporal progression of the DDR, Kieffer and Lowndes propose that the response to DSBs can be divided into immediate-early, early, and late responses, in analogy to the events occurring upon viral infection. Their review provides an integrated view of these sequential DDR responses and how they are modulated by the complexity of the DSB end, chromatin context, cell cycle phase, and the availability of specific DSB repair factors to control DSB repair pathway choice.

The packaging of DNA into chromatin, the so-called ‘chromatin barrier’, complicates the efficient detection and repair of DSBs (Goodarzi and Jeggo, 2012). ATP-dependent chromatin remodelers and post-translational modifications (PTMs) of histones and other chromatin-associated proteins are therefore required to modulate chromatin structure around DSBs and facilitate repair. The review by Karl et al. covers the latest insights into the function of several chromatin remodelers and their impact on DNA end-resection, which is a critical determinant of DSB repair pathway choice. The authors describe recent advances in understanding the role of nucleosome sliding and positioning, editing, and eviction on resection and DSB repair.

Several mechanisms ensure that HR is restricted to the S and G2 phases of the cell cycle, including the antagonism between the DSB-responsive chromatin readers 53BP1 and BRCA1 (Hustedt and Durocher, 2016). The review by Sanchez et al. covers the latest insights on the diverse nature of protein interaction domains involved in the DDR, their crosstalk within chromatin, and how multiple, sometimes competing signals are integrated at the level of the chromatin scaffold for proper DSB repair. Further strengthening the role of chromatin structure and nuclear topology for repair, the review by Sebastian et al. describes the processes that shape the three-dimensional (3D) chromatin landscape and how they impact genome functions including DNA replication and DSB repair. Besides chromatin context and topology, DSB movement into repair-permissive environments and the potential role of phase separation are discussed. A particular challenge for DSB repair is posed by dense heterochromatin, and recent studies have revealed how heterochromatic features influence DSB repair. The review by Caron et al. covers the latest insights on this topic and discusses the interplay between heterochromatin marks and DSB repair, focusing on the role of both pre-existing heterochromatin domains and *de novo* establishment of heterochromatin features in euchromatic regions upon DNA damage.

Despite recent technical improvements, studying chromatin structure and dynamics at high spatial and temporal resolution remains challenging. The research article by Lou et al. describes a novel approach to look at nanoscale chromatin changes based on fluorescence lifetime imaging microscopy (FLIM) of Förster resonance energy transfer (FRET) between fluorescently labeled histones. Employing the DSB-inducible AsiSI cell system (DIvA), their approach has sufficient spatial resolution to map chromatin compaction nuclear-wide and the authors use this to elucidate how nanoscale chromatin architecture impacts the balance between competing DSB repair pathways such as NHEJ and HR.

According to current models, HR repair comprises DNA end-resection followed by homology search (Wright et al., 2018). Once homology is found, usually on the undamaged sister chromatin, a displacement loop (D-loop) is formed which allows DNA repair synthesis. However, after DNA repair synthesis is complete, HR can proceed via different HR sub-pathways. The review by Elbakry and Löbrich highlights these alternative sub-pathways, including the canonical sub-pathways of synthesis-dependent strand annealing (SDSA) and the Holliday junction (HJ) pathway, as well as the non-canonical break-induced replication (BIR) pathway, and discusses clinical implications of HR sub-pathway choice.

A central protein in the orchestration of HR is the tumor suppressor BRCA2. Mutations in the *BRCA2* gene are associated with breast and ovarian cancer, but how individual *BRCA2* mutations affect HR is incompletely understood. The research article by Jimenez-Sainz et al. sheds light on this issue by revealing that the pathogenic variant R3052W causes mis-localization of BRCA2 to the cytoplasm. The defect in nuclear localization can thus explain the HR deficiency, which results in genome instability and sensitization to PARP inhibitors and crosslinking drugs.

Besides gene mutations, changes in expression of DNA repair genes frequently contribute to tumor formation. In recent years it has become clear that tumors can reactivate genes whose expression is normally restricted to germ cells. The review by Lingg et al. discusses the function of meiotic genes and how their aberrant reactivation in somatic cancer cells affects DSB repair and genome stability. Considering that meiotic genes are transcriptionally repressed in somatic cells of healthy tissues, targeting reactivated meiotic genes could provide a therapeutic opportunity to specifically kill cancer cells.

The ability of cells to proliferate depends on the faithful duplication of their genome via DNA replication during S phase of each cell cycle. Upon replication stress, cells coordinate a variety of genome and cell cycle surveillance pathways to ensure the completion of replication and maintain genome stability (Panagopoulos and Altmeyer, 2021; Saxena and Zou, 2022). The review by Wootton and Soutoglou provides an overview on the many aspects of chromatin and nuclear environment such as topologically associated domains (TADs), non-canonical histone variants, and histone modifications, and how these affect replication fork stability, S-phase progression and repair of replication-associated DNA damage. Extending this theme, the review by Nickoloff et al. focusses on the safe and unsafe pathways to repair broken replication forks, highlighting the danger of erroneous single-ended DSB repair by NHEJ, and describing mechanisms to ensure that broken forks are instead repaired faithfully by HR.

For most two-ended DSBs, however, NHEJ seems to be the predominant or fastest repair pathway in mammalian cells. This type of end-joining repair also plays an important role during V(D)J recombination, which occurs during lymphocyte differentiation to generate antibody diversity. During this process DSBs are introduced by the RAG nuclease, and the review by Libri et al. describes various parameters that constrain the repair of RAG-induced DSBs to NHEJ, including DSB-end structure, the presence of a post-synaptic cleavage complex, and protection against DSB end resection.

This Research Topic also features articles that discuss newly emerging methodologies to investigate how cells commit to a certain repair pathway. The review by Meyenberg et al. provides a comprehensive overview on recent developments in the context of tissue specific DNA repair upon CRISPR-induced DNA breaks. The authors also discuss the implications for genome editing and gene therapies to treat genetic diseases. Extending on the CRISPR methodology, the review by van de Kooij and van Attikum describes the advent of Cas9 nucleases in the construction of novel reporter systems to measure DSB-repair pathway usage. They compare single-pathway and multi-pathway DSB-repair reporters and highlight how the new Cas9-based reporter systems enhance the flexibility and design of reporter constructs in comparison to established I-SceI reporter systems. Finally, the methods article by Schep et al. provides a detailed protocol for DSB-TRIP, a technique that utilizes genomic scars left behind by DNA repair to study DSB repair pathway usage throughout the genome and correlate repair pathway choice with various chromatin features.

CRISPR-based screens have greatly facilitated the identification of synthetic lethal interactions relevant to DNA repair and replication in normal and cancer cells (Setton et al., 2021; Wilson and Loizou, 2022). Synthetic lethality, or sickness, describes a cellular condition in which a defect in either one of two genes has little or no effect on cellular fitness, whereas the combination of both gene defects results in cell death or severely compromised fitness, respectively (Setton et al., 2021). The review by Rossi et al. highlights recent studies on the importance of the repair protein RAD52 to keep HR-deficient cancer cells viable. The critical role of RAD52 in this context makes it an attractive target for the development of anti-cancer therapies to treat HR-deficient tumors. Apart from such targeted therapeutic approaches based on the concept of synthetic lethality, radiotherapy is widely used for the treatment of tumors, and particularly particle radiotherapy has received increasing attention due to dose distribution advantages. The review by van de Kamp et al. describes different types of ionizing radiation in the context of radiotherapy, and discusses the DNA lesions they induce and how these in turn impact DNA end processing and repair. Moreover, combination therapies and promising DDR targets that could improve particle radiotherapy are discussed.

Together, this article collection highlights the growing understanding of the fundamental principles of DNA repair pathways and their context-dependent regulation. At the same time, the collection also sheds light on the many unknowns that still exist about repair pathway and sub-pathway choice in different biological settings and disease conditions. Future research and emerging technologies, some of which are described in this collection, will aim at turning these insufficiently understood areas into new knowledge that can be used to harness DNA repair for targeted genome editing and precision cancer therapy to improve clinical outcomes in patients.
